# SWOT Analysis and Recommendations for Community Health Workers and Stakeholders Responding to COVID-19 Health Inequities

**DOI:** 10.1177/15248399231201131

**Published:** 2023-10-17

**Authors:** I. Niles Zoschke, Alejandro Betancur, Sara Ehsan, Jill D. TenHaken, Justin R. Rahman, Kim King-Tezino, Megan Kramer-Najjar, Carlos A. Bravo, J. Michael Wilkerson

**Affiliations:** 1 University of Texas Health Science Center at Houston, Houston, TX, USA; 2 Harris County Public Health, Houston, TX, USA

**Keywords:** career development/professional preparation, lay health advisors/community health workers, partnerships/coalitions, formative evaluation, program planning and evaluation, workforce development, health equity

## Abstract

*Background.* By 2023, 1,080,000 cases of COVID-19 have been reported in Harris County. Systemic inequity and vaccine hesitancy have contributed to COVID-19 disparities. Community Health Workers provide health education and instrumental support to alleviate health disparities among vulnerable communities. We conducted an analysis of Strengths, Weaknesses, Opportunities, and Threats (SWOT) analysis in June 2022 among a broad coalition of Community Health Work stakeholders to better understand the local landscape in the context of the COVID-19 pandemic. *Methods.* We recruited 33 community health workers and industry stakeholders in Harris County, Texas, to participate in the SWOT analysis. Participants were asked to describe their opinions on the SWOT facing the Community Health Work landscape and then rank the outcomes of the analysis to prioritize action. *Results.* A total of 19 themes were identified. Weaknesses included lack of respect and resources for Community Health Workers and poor coordination and capacity among the workforce infrastructure. Limited funding and lack of appreciation for Community Health Workers were deemed important threats. Diversity and community connection were critical strengths, and strong education, training, and raising awareness for community health work were considered opportunities to overcome identified weaknesses and threats. *Discussion.* Increased funding, greater coordination, greater respect, and amplified training can improve capacity for Community Health Workers and, therefore, improve public health outcomes for respiratory illness and viral infections such as COVID-19. This analysis helps fill an important research gap on the topic Community Health Workers responding to public health crises with racially disparate outcomes.

By 2022, the national COVID-19 mortality rate for Black Americans was 2.3 times higher than White Americans (Xu et al., 2022). In Texas, Black and Hispanic residents experience higher rates of COVID-19 infection, are more likely to die from the disease, and experience higher economic burden from the pandemic (Xu et al., 2022). More than 1,080,000 cases of COVID-19 and 8,600 COVID-19-related deaths have been reported in Harris County, Texas, the county where the city of Houston resides (Harris County Public Health [HCPH], 2023). By March 2023, non-Hispanic Black Harris County residents experienced substantially lower cumulative vaccination rates compared with non-Hispanic White residents (OR = 0.71; CI = 0.71–0.72), likely due to lower access to vaccines, protective equipment, and health care, as well as overall racial and ethnic income inequality (HCPH, 2023; Xu et al., 2022).

Harris County Public Health and local community-based organizations employ Community Health Workers (CHWs; CHW for singular) to support vulnerable communities in vaccine access and informational updates. The American Public Health Association defines a CHW as “a frontline public health worker who is a trusted member of and/or has an unusually close understanding of the community served” and continues, by stating, “This trusting relationship enables the worker to serve as a liaison/link/intermediary between health/social services and the community to facilitate access to services and improve the quality and cultural competence of service delivery (APHA.org, n.d.).”

A CHW also builds individual and community capacity by increasing health knowledge and self-sufficiency among community members through a range of activities such as outreach, community education, informal counseling, social support, and advocacy, and directly addresses issues which impede effective COVID-19 prevention (Parekh et al., 2019). CHWs have also been employed in asthma and chronic obstructive pulmonary disease management, two common respiratory diseases (Coutinho et al., 2020; Fox et al., 2007; Martin et al., 2021; Parekh et al., 2019). A 2020 systematic review and other recent research demonstrated that CHWs are effective in treating and preventing respiratory diseases, especially among children with asthma, but may be underutilized in assisting adult populations (Coutinho et al., 2020; Fox et al., 2007; Martin et al., 2021; Parekh et al., 2019). Despite the critical role CHWs fill in providing essential public health services, there is a lack of published research on the topic of CHWs responding to public health crises, on cost-effective reimbursement for services, and on training CHWs to conduct public health research interventions (Coutinho et al., 2020; Nebeker et al., 2021).

In this article, we report the outcomes of a formative evaluation conducted with HCPH on a growing CHW coalition, using narratives of CHWs and their leaders. This research helps expand upon recent, yet limited research describing the value of CHWs in their own words (Nebeker et al., 2021). In this article, we also provide important context from contemporary scholarly literature and make recommendations to stakeholders employing CHWs in similar contexts for improving CHW training and support infrastructure.

## Method

This project was a formative evaluation taking a participatory action approach by conducting a strengths, weaknesses, opportunities, and threats (SWOT) analysis among organizations working with or employing CHWs in Harris County in 2022. A SWOT analysis is a participatory action analysis that invites community partners to identify the internal and external factors, which help or hinder the organizations from achieving the desired outcomes (Renault, n.d.). The internal factors (*strengths* and *weaknesses*) are the aspects within each organization or community that facilitate or impede success, whereas the external factors (*opportunities* and *threats*) are contingencies beyond the direct control of the community and organizations which can influence outcomes. The evaluation team consisted of program evaluators from The University of Texas Health Science Center at Houston (UTHealth) and CHW program implementers at HCPH. The purpose of this SWOT analysis was to identify the *strengths* and *weaknesses* of existing CHW coalitions, and other CHW-focused organizations in the region, as well as to identify *opportunities* and *threats* necessary to overcome.

The evaluation team derived the SWOT protocol from Chapter 3 of the Community Tool Box (Renault, n.d.). The evaluation team split the SWOT analysis into three teleconference sessions with each session lasting around 2 hours, spread over 3 consecutive weeks, to avoid fatiguing participants and to give us time to process the data for the following session. Harris County Public Health leveraged their relationships to recruit participants from CHW stakeholder organizations, training centers, research organizations, and active work groups. Recruitment communications requested that each organization send one supervisor and one CHW to the SWOT activity to diversify and balance perspectives. Prior to commencing the SWOT analysis, all participants signed an informed consent approved by the UTHealth Committee for the Protection of Human Subjects (institutional review board [IRB]). All participants filled out a survey delivered via REDCap, capturing basic demographic data prior to participation.

The objective of the first session was to ask the participants to list and discuss the elements of the SWOT. The evaluation team stated the objectives of the analysis and facilitated a space for the group to establish its own rules and norms of conduct. The evaluation team drew this approach for setting ground rules from the University of Minnesota Extension (Hinz, n.d.). The evaluation team also taught the participants how to use Jamboard, an online, collaborative presentation application that allows participants to post comments (Google, n.d.). Afterward, the evaluation team divided participants into three separate breakout groups (maximum eight people per group), each facilitated by a member of the evaluation team. The evaluation team was careful to place participants from the same organization into different subgroups, and to mix CHWs with supervisors to balance power and allow for participants to be honest in their discussion about all aspects of SWOT.

In the breakout groups, the participants were asked about the SWOT of the local CHW infrastructure, in light of the COVID-19 pandemic. The participants then wrote their comments on Jamboard, while a member of the evaluation team facilitated discussion. After the breakout sessions concluded, a representative from each breakout group volunteered to present the findings to the larger group. Finally, the evaluation team opened the floor for all the participants to discuss the identified SWOT. This concluded the first session.

The evaluation team then collated the comments and discussion generated during SWOT Session 1. Two evaluation team coordinators and one research assistant manually qualitatively coded the data and themed the data by consensus, looking for commonalities, patterns, exceptional cases, and divergences in data (Tolley et al., 2016). One of the research coordinators had an extensive background in qualitative analysis and participatory action analysis and guided this process. During SWOT Session 2, the evaluation team presented these themes to the participants with the objective of setting priorities using the *relative worth* method for decision-making, drawn from Community Tool Box (Rabinowitz, n.d.). For this activity, each participant had three votes per SWOT category and applied the votes to indicate which themes were most important and which themes were most likely to change. There were two rounds of voting; the first asked participants to indicate which items are the most important. The second round of voting asked the participants to indicate which items were more prone to change in the short or intermediate term. This concluded SWOT Session 2.

The evaluation team again tallied the votes and then presented the results to the attendees during SWOT Session 3 while facilitating discussion and interpretation of the findings to conclude the SWOT analysis exercise.

## Results

### Demographics

A total of 29 unique individuals participated in both data gathering Sessions 1 and 2. An additional four people participated in the final session where the evaluation team reported the results back to the participants. A total of 33 unique people attended any of the three sessions. Participants were racially and ethnically diverse and mostly women, both new and seasoned in the field. The majority of participants had a graduate school degree. Nine participants were CHWs, ten were CHW supervisors, eight were CHW project leads, and ten identified as “something else,” for example grant writers, recovery coaches, and equity officers. It is important to note that these responses were not mutually exclusive, and in two cases, a participant responded with multiple roles. Participants worked in agencies addressing a range of social determinants of health, including access to health care, food security, and housing.

### SWOT Outcomes

We listed the responses and emergent themes for internal SWOT factors in Table 1, and the themes for the external SWOT factors in Table 2. We applied the outcomes of the ranking exercise and discussions to generate “heat maps,” or tables that demonstrate the tallies of how participants voted for each theme across both importance and changeability. Tables 3 and 4 display these results and context drawn from conversations in each session for the SWOT internal and external factors. Themes highlighted in green had a combined score of 0 to 10; yellow, 10 to 20; orange, 20 to 30; and red, 30+. Themes in green are ranked as low priority, yellow as moderate priority, orange as high priority, and red as the highest priority. Priority was ranked from highest to lowest combined importance and changeability scores.

**Table 1:** CHW Landscape Internal Factors Theme Report

Emergent theme (*Strengths*)	Jamboard responses which comprise associated theme, verbatim
Support to CHWs to help the community	Major partners providing resources to CHWs, such as masks, educational materials, and technology to work from home.
	Connections to other organizations and services to meet needs
	Good support system
	CHWs are taking the lead and participating in what the community needs, especially during the pandemic.
	CHWs listen to the community and provide what is needed.
Connection to the community	Direct communication with community during pandemic
	The community is like home to the CHW—they are part of the community, or it is similar to what they know or they have worked there a long time.
	Truly want what’s best for the community
CHWs are highly diverse, competent, flexible, and available part of the public health workforce	CHWs are competent in teaching patients how to access virtual health.
	CHWs reduce travel time for patients.
	CHWs are flexible, for example, they can work from home, or work in person, or switch back and forth using the necessary technology and safety measures.
	CHWs come from diverse backgrounds and are rooted in the community.
	CHWs have lived, hands-on experience.
There are ample job opportunities for CHWs	CHW community is empowered by ample job opportunities.
	COVID-19 provided new opportunities for CHWs and boosted their national recognition.
	Too many jobs are not filled.
	At least one organization was able to maintain a full CHW workforce without any layoffs during the pandemic.
There are ample training opportunities for CHWs	Texas certification program
	Education provided to CHWs—weekly webinar about the crisis and situation, community needs and challenges, hotspots, mental health first aid.
	Core competencies and certification give credibility for research, health care, and wellness programs.
	Training focus on self-development, and it is useful on a professional and personal level.
	Give CHWs easy tools and explain to community in lay language
The role of CHWs is respected among institutions	CHWs are considered a best practice.
	Respect and recognition garnered in their department or among supervisors.
	CHWs integrated into the four pillars of the Harris County Health System.
	Many organizations know and respect CHWs.
Emergent theme (Weaknesses)	Jamboard responses which comprise associated theme, verbatim
Public health system perceived as fragmented, especially in light of the pandemic, and CHWs are expected to fill the gaps	COVID interrupted sense of CHW mission and has made the system fragile/fragmented.
	Fragmented CHW infrastructure, lack of cohesiveness, working in silos
	Many health agencies shut down during the pandemic.
	CHWs are treated as band-aids to a fractured public health system.
	Lack of community-driven accountability
Emergent theme (*Weaknesses*)	Jamboard responses which comprise associated theme
CHWs are misunderstood, marginalized, disrespected, under-resourced, and underpaid	Many stakeholders including clinical staff do not understand the role of a CHW
	Lack of respect for CHW
	We’re not provided the same personal protective equipment that clinical staff were
	CHWs are high value but get paid low wages.
	The expectations for CHWs are unrealistic.
	Academic institution and not as a health institution took executive/administrative decisions that excluded CHWs and did not value the role of the CHWs or allow them to work.
	Large workforce that still does not have clarity as to CHW roles and functions
	Limited time to brief the clinical staff on CHW roles and responsibilities
	Sometimes, immediate supervisors do not understand the role and functions of CHWs.
	CHWs are photo-ops but not respected.
	Clinical staff do not understand or are hesitant to tackle social determinants of health.
	IRB and perhaps health institutions wary of offering too much in terms of social services that they may not be able to provide—Health institutions did not know how to direct patients to community resources.
	Status or education (PhD vs. other; “expertise”)
	Dismissive of CHWs during pandemic
	Clinicians do not understand or respect if you do not speak the clinical lingo.
CHW training needs further definition, development, and promotion	CHW certification training has no classification.
	Need more marketing for CHW training and educational opportunities
	Training programs should be streamlined and more collaborative
	Not producing prosperous, happy CHWs
CHW stakeholders need increased capacity, coordination, planning, and communication	Communication gap among CHWs due to lack of communication platforms
	Lack of advertising/recognition of CHWs and their role in the community
	Lack of database/meetings in the CHW realm to share information and experience
	Lack of capacity-building programs form CHW infrastructure
	Lack of vision and goals for CHW infrastructure
	Poor coordination between networks/organizations, which serves institutional agendas
	Planning is not based in the community but rather in institutions.
The CHW workforce needs further development and support	Lack of jobs
	Lack of diversity in job opportunities
	Work instability
	Not enough CHWs
	Stakeholders do not always know how to support CHWs
	How do you put a dollar amount on love for the community?
	Bilingual CHWs are preferred—more difficult for monolingual CHWs.
	Inadequate pay according to job prerequisites
	Systemic racism is not being addressed in hiring practices.
	Difficulty transitioning to COVID navigation
	Higher thresholds to become CHW

*Note.* CHW = Community Health Worker; IRB = institutional review board.

**Table 2:** CHW Landscape External Factors Theme Report

Emergent theme (*Opportunities*)	Jamboard responses which comprise associated theme, verbatim
Education and training opportunities can be improved to provide specific skills and competencies needed to the workforce	Lots of educational and training opportunities, including ones that are bicultural and bilingual. These can be improved and developed further by specializing on specific skills for certain jobs, such as cultural competency, and curriculum on managing chronic disease and crisis management.
	Enhancing training on a local level
	Offering specializations in CHW training and classifying the workforce
	Hiring more Spanish and Vietnamese-speaking CHWs and bridging the language barrier.
	Offering incentives to CHWs for participating in training programs
	Increasing hands-on training
	Utilizing experienced CHWs to train new ones in various roles
Building recognition for CHW can increase value for the field	Representation of CHWs as stakeholders
	Getting Governor’s Proclamation annually to build recognition
	CHWs are getting more recognition now both in the community and in public health research.
Increasing collective ownership and power for CHWs to build a sustainable framework	A capacity-building component and a long-term framework required
	Building power for CHWs
	Increased collective ownership for CHWs
	Secure funding to adequately pay CHWs
Increasing coordination among partners, especially for sharing resources and supporting CHWs	Establishing a clear communication structure
	Building trust between community and health care providers
	Establishing a system to connect CHWs to jobs, for example, a resource fair to educate the community on opportunities for CHWs
	To establish system cohesiveness, bringing all stakeholders together under one umbrella (communication platform)
	Working with and learning from partners in CHW task forces
	Instead of working in silos, work together to improve the community and CHWs
	New CHW task force
	More support for job title
Emergent theme (Threats)	Jamboard responses which comprise associated theme, verbatim
Large organizations want to control CHWs but do not fully appreciate their benefits	Large institutions dominate and control CHW
	Health care system missing the benefits of CHWs
	CHWs are exploited.
	Organizational agendas taking over and people are unable to work together.
	CHWs do not understand or want to understand or reject corporate jargon/roles.
Limited funding	Funding for CHWs is limited and short term.
	Lack of support for CHW professional growth when no longer needed or funding ends
Academic qualifications and linguistic preferences	“Professionalizing” CHW could reduce workforce and makes it more difficult for advance.
	Academic qualifications and linguistic preferences are desired and have an impact on how highly compensated and quickly employed a CHW is
	Even though they are supposed to hold a bachelor’s degree, CHWs are paid very little (not enough to pay-off student loans)
CHW landscape is shifting and unstable	An efficient CHW infrastructure has existed at various time points in the past owing to the efforts of passionate individuals who believe in the workforce, but when they leave or become involved in other activities, the system falls apart. Fragility and lack of sustainable framework.

*Note.* CHW = Community Health Worker.

**Table 3:** CHW Landscape Internal Factors Priority Heat Map

Label	*Strengths*	Importance	Changeability	Combined score	Context drawn from original commentary
S1	CHWs are highly diverse, competent, flexible, and available part of the public health workforce	24	7	31	Comments mostly related to their flexibility and being able to work from home during the pandemic. There were several comments about the diversity of the CHWs and their being rooted in the community. The CHWs have hands-on experience.
S2	There are ample training opportunities for CHWs	9	19	28	Comments highlighted the certification process which lends credibility and develops competencies among CHWs. This certification focuses on self-development and leadership skills. Training opportunities cover updates on COVID hotspots, mental health first aid, and community needs and challenges.
S3	Connection to the community	18	5	23	These comments refer to the interactions between the CHW and the community they work in. CHWs listen to what the community needs. They engage directly with the community. They are usually from or very familiar with the communities they serve (they have worked there a long time). They want what’s best for the community.
S4	Support to CHWs to help the community	3	10	13	These comments refer to the emotional support CHWs receive from each other and from their families to do the work they do. It also includes comments on connections with other organizations that provide CHWs with resources and technology. It also refers to connections to organizations that connect CHW's clients to services.
S5	The role of CHWs is respected among institutions	9	4	13	Among participants, there are two different perspectives on this theme, which is why it is listed as a *strength* and a *weakness*. The comments that comprise this theme refer to CHWs being a best practice among organizations. They also refer to their work being recognized by supervisors, departments, and organizations.
S6	There are ample job opportunities for CHWs	0	4	4	Some comments focused on unfilled jobs while others highlighted maintaining CHW staff throughout the pandemic by securing external funding. Other comments focused on CHWs feeling empowered by ample job opportunities.
Label	*Weaknesses*	Importance	Changeability	Combined Score	Context drawn from original commentary
W1	CHWs are misunderstood, marginalized, disrespected, under-resourced, and underpaid	26	3	29	This theme is comprised of comments about: Systemic racism The underemployment of black women Bad pay or no pay at all, and wholesale exploitation Misunderstanding of CHW roles and functions among supervisors and organizations Lack of support, such as COVID protective equipment Tokenization via photo-ops Not being recognized as integral parts of the workforce Hierarchy based on educational status and language, such as mastery of medical jargon
W2	CHW stakeholders need increased capacity, coordination, planning, and communication	13	11	24	Commentary stated that organizations and networks that employ CHWs do not communicate or check-in with each other. Currently, there is no place to share information and experiences. In addition, there is a lack of vision and goals for infrastructure development.
W3	The CHW workforce needs further development and support	11	6	17	Participants cited lack of jobs, lack of diversity, systemic racism, and low numbers of CHWs for this theme.
W4	CHW training needs further definition, development, and promotion	2	11	13	The comments comprising this theme stated CHW-based organizations need marketing to advertise training opportunities. In addition, stated was that training should be streamlined and made more collaborative, and that currently, the CHW ecosystem lacks capacity-building programs.
W5	Public health system perceived as fragmented, especially considering the pandemic, and CHWs are expected to fill the gaps	11	0	11	These comments refer to the fragmented nature of the CHW infrastructure. For example, there is a lack of cohesion as many people and organizations work in silos. It was stated that CHWs are often called in as an afterthought while the public health system is falling apart. In addition, it was stated that many organizations are unwilling to address the social determinants of health.

*Note.* CHW = Community Health Worker.

**Table 4:** CHW External Factors Priority Heat Map

Label	*Opportunities*	Importance	Changeability	Combined Score	Context drawn from original commentary
O1	Education and training opportunities can be improved to provide specific skills and competencies needed to the workforce	15	19	34	The associated comments for this theme focus on ways that training can be improved. For example, training can be improved by offering incentives, providing training on crisis management and cultural competency, increasing hands-on experience, utilizing experienced CHWs for training facilitation, and increasing the number of trainings offered locally. One participant remarked that improving training would a trivially easy change.
O2	Increasing coordination among partners, especially for sharing resources and supporting CHWs	19	15	34	For this theme, there were many comments related to communication and networking. These included the need to: • Build a communication structure • Bring stakeholders together to increase cohesion • Learn from other CHW task forces nationally • Work together and not in silos • Connect CHWs to employment (e.g., hosting resource fairs) • Build trust between the community and health care providers
O3	Building recognition for CHW can increase value for the field	17	15	32	This theme refers to recognition such as increased community respect and interest in public health, for example, the Governor’s Proclamation helped to build recognition.
O4	Increasing collective ownership and power for CHWs to build a sustainable framework	13	4	17	The associated comments for this theme refer to building power for CHWs. One suggested method is increased collective ownership for CHWs. The existing CHW infrastructure exists, and is efficient and successful, because the efforts of people who believe in the power of CHWs. However, when champions leave the CHW ecosystem, the ecosystem can collapse because no one is left to guide and sustain it over the long term.
Label	Threats	Importance	Changeability	Combined Score	Context drawn from original commentary
T1	Limited funding	29	21	50	Funding was stated to be short term.
T2	Large organizations control CWHs but do not fully appreciate their benefits	21	6	27	Comments articulated that most planning is organization-based rather than community-based.
T3	CHW landscape is shifting and unstable	7	5	12	The concerns stated that the ecosystem is not applying systemic approaches to public health concerns, and that the infrastructure is built up by passionate people, who, when they leave, can cause the system to fall apart.
T4	Academic qualifications and linguistic preferences	5	1	6	The problem cited here is the professionalization of CHWs which makes it more difficult to get jobs.

*Note.* CHW = Community Health Worker.

We took the results from the heat map and cross-tabulated the “hottest” themes to connect *strengths* with *weaknesses*, *opportunities*, and *threats*, thematically, for practitioners to consider as they implement the knowledge gained from the SWOT analysis. For example, we aligned “hot” factors surrounding training across *strengths*, *weaknesses*, and *opportunities*, to highlight how these themes connect. These areas serve as opportunities for CHW-focused organizations to take immediate action. Table 5 displays this cross-tabulation.

**Table 5:** Cross-Tabulation of Hot Factors

Strengths	Weaknesses	Opportunities	Threats
(S1) CHWs are highly diverse, competent, flexible, and available part of the public health workforce.	(W1) CHWs are misunderstood, marginalized, disrespected, under-resourced, and underpaid.	(O1) Education and training opportunities can be improved to provide specific skills and competencies needed to the workforce.	(T2) Large organizations control CWHs but do not fully appreciate their benefits.
(S3) Connection to the community	(W1) CHWs are misunderstood, marginalized, disrespected, under-resourced, and underpaid.	(O3) Building recognition for CHW can increase value for the field.	(T2) Large organizations control CWHs but do not fully appreciate their benefits.
—	(W2) CHW stakeholders need increased capacity, coordination, planning, and communication.	(O2) Increasing coordination among partners, especially for sharing resources and supporting CHWs	(T1) Limited funding
(S3) Connection to the community where the community resides	(W2) CHW stakeholders need increased capacity, coordination, planning, and communication.	(O2) Building recognition for CHW can increase value for the field.	(T1) Limited funding
(S2) There are ample training opportunities for CHWs.	(W4) CHW training needs further definition, development, and promotion.	(O1) Education and training opportunities can be improved to provide specific skills and competencies needed to the workforce.	(T2) Large organizations control CWHs but do not fully appreciate their benefits.

*Note.* CHW = Community Health Worker.

## Discussion

The results of the SWOT analysis correspond to and expand on existing literature on the importance of employing CHWs for community health issues, as well as the public health infrastructure’s response to the COVID-19 pandemic. This research responds to a recent call for more research on how CHWs are employed in preventing and managing respiratory diseases (Parekh et al., 2019). While this study was not an empirical or outcome evaluation of a specific CHW-based intervention, researchers and practitioners can apply the outcomes of this SWOT analysis to inform regional approaches employing CHWs responding to public health crises.

While CHWs may be key players in the management of respiratory diseases like COVID-19, structural difficulties in the public health system impeded their work. During the SWOT analysis, one participant commented “CHWs are treated as band-aids to a fractured public health system.” This comment validated literature surrounding public health service fragmentation, exacerbated by the intense stress of the COVID-19 pandemic. The evaluation team developed the following recommendations to assist public health agencies to effectively employ CHWs in the context of a public health crisis, by collaborating with one another, increasing sustainability for community health work, elevating the role of CHWs institutionally and within the community, and building CHW capacity to serve.

### Recommendation #1: Gather Data and Incorporate Findings Into Partnership-Wide Strategic Planning and policy Advocacy

In the following sections, we will refer to the various *SWOT* using letters and numbers we assigned to them in Tables 3 to 5. For example, *Weakness 1* is known as W1. One such *weakness* identified in the SWOT analysis was *CHW stakeholders need increased capacity, coordination, planning, and communication* (W2). To overcome this weakness, practitioners and researchers can apply the results from the present analysis to improve planning and to conceptualize more specific, limited, and strategic evaluation questions to close gaps in knowledge. For example, stakeholders can survey agencies about their activities and available resources to learn how to streamline services. Furthermore, stakeholders can involve policy champions in data collection and analysis to set policy agendas which are local, specific, and relevant to the conditions which CHWs face (Sabo et al., 2017). Researchers investigating policies to strengthen CHW infrastructure are challenged by tremendous variability between CHW ecosystems, as well as by shifting public health conditions. Therefore, gathering more local data from CHWs and CHW stakeholders on ideal policies is necessary for strategic planning (Sabo et al., 2017). Practitioners can combine the results of this analysis with other data to fuel collaborative deliberation among all stakeholders to best translate outcomes into effective action.

### Recommendation #2: Increase Coordination, Cohesion, and Trust Among CHW Stakeholders

The *weakness* W2, *CHW stakeholders need increased capacity, coordination, planning, and communication*, corresponds to poor coordination and cooperation among the CHW stakeholder organizations. Community participants stated that at the time of analysis, there was no apparatus or space in place to share information, experiences, or resources. In addition, community participants stated there was no clear, shared, vision or goals between CHW stakeholder organizations to develop CHW infrastructure. Community participants stated that poor coordination contributed to another *weakness*, W5, *Public health system perceived as fragmented, especially considering the pandemic, and CHWs are expected to fill the gaps*. Community participants stated that organizations worked in silos due to lack of cohesion, and that CHWs were an afterthought in a crumbling public health system, exacerbated by the COVID-19 pandemic.

However, the aforementioned *weaknesses* directly correspond to the identified *opportunity* O2, *Increasing coordination among partners, especially for sharing resources and supporting CHWs*. According to community participants, there was a major opportunity to (1) increase the coordination and cohesion among partners, (2) share resources and support for CHWs by building a common infrastructure, (3) learn from other national CHW task forces, (4) connect CHWs to sustainable employment rather than short-term “band-aid” approaches to public health challenges, and (5) ultimately, build trust between health care providers and the community to improve health outcomes and mend the broken public health system.

To respond to these concerns, CHW stakeholders can align services and increase cooperation. While every local organization involving CHWs can play a role in building this common infrastructure, local and county government health agencies appear well suited to build on existing work, especially in partnership with academic institutions, such as that of the CHW Core Consensus (Rosenthal et al., 2021). As recommended by a SWOT participant, local efforts can also look to other states’ work for guidance. For example, the Department of Health in South Dakota implemented a series of events and strategies throughout the year to unify the CHW landscape, such as convening monthly CHW trainings, hosting educational conferences, and disseminating technical support newsletters for CHWs (Community Health Workers Collaborative of South Dakota, 2022). To build on this established strategy, partners in Harris County can host similar events and develop communications to strengthen regional action. Future partnership-wide strategic planning serves as a tremendous opportunity to implement these recommendations.

### Recommendation #3: Improve Sustainability for CHWs by Increasing Renewable Funding

The *threat* identified by community stakeholders with the highest combined importance and changeability scores was T1, *Limited funding*. During the SWOT analysis, community participants stated that funding was limited and short-term, and when funding ended, professional support for CHW also ceased. This result corresponds to the findings of a systematic review of community health work conducted in Hawai’i, which revealed that CHW jobs are funded by a patchwork of grants that face challenges with reliable service reimbursement from funders (Stupplebeen et al., 2019). The identified *threat* T1, *Limited funding*, also closely corresponds with findings from a recent policy review which indicated that funding instability threatened CHW program sustainability, and, therefore, weakened CHW capacity to provide essential services to communities during the COVID-19 pandemic (Schmit et al., 2022).

Schmit et al. (2022) concluded that CHW stakeholders should clarify the roles and definitions of CHWs to educate funders on how CHWs fit into programmatic goals. Furthermore, Alvillar et al. (2011) suggested educating employers about the evidence of CHW cost-effectiveness can encourage employers to hire and sustain CHWs. Another way to help program sustainability is to advocate for Medicaid, Medicare, and/or State Children’s Health Insurance Program funds reimbursement to pay CHW salaries. Successful advocacy will require a collaborative, statewide effort including policy champions from nonprofits, hospitals, and state institutions. The findings in the aforementioned policy and practice analyses directly correspond with our findings in the *opportunity* O3, *Building recognition for CHW can increase value for the field*, and the *weakness* W1, *CHWs are misunderstood, marginalized, disrespected, under-resourced, and underpaid*. The recommendations listed here can capitalize on the *opportunity* O3 to overcome the *weakness* W1.

### Recommendation #4: Raise Wages and Improve Dignity for CHWs

Despite CHWs’ critical role in public health infrastructure, and the express need for their labor, community participants reported that wages, respect, and opportunity for advancement were low. These concerns correspond with the identified *weakness* W1, and *CHWs are misunderstood, marginalized, disrespected, under-resourced, and underpaid*. Community participants stated that due to a lack of understanding of the role of CHWs, or even wholesale exploitation, CHWs were not respected as integral workers in public health. Community participants were concerned that systemic racism contributed to the condition where the labor of Black CHW women was taken for granted or even tokenized in photograph opportunities with little respect otherwise.

Implementing *Recommendation #3, Increase sustainability for CHWs by increasing renewable funding* can improve poor working conditions and low wages by communicating the value of CHWs to institutional stakeholders. It can also help by increasing employment sustainability, ensuring CHWs are consistently paid, by providing opportunities for professional development, and by developing novel approaches to increase funding. However, we also recommend that organizations employing CHWs examine and address their potential implicit biases. Such organizations can conduct the Implicit Association Test within their organizations and apply the findings to reduce discrimination (Greenwald et al., 2022). We also strongly recommend organizations deeply consider ways in which they can move beyond mere tokenization of CHWs and avoid relying on CHWs as fix-all solutions to systemic problems. Rather, we recommend incorporating their voices into decision-making, with a commensurate increase in wages and respect. One way to develop the role for, and capacity of, CHWs, is to train them to effectively respond to deeply rooted public health issues, and to train them as leaders in the field.

### Recommendation #5: Train CHWs in Collaboration With Partners and Further Define the Scope of Practice

The importance of training and education was another recurrent theme that emerged from the SWOT analysis. Training is a multifaceted activity that ideally allows participants to debrief on work experiences, network, connect, and talk. It is also a means to develop workers and improve working conditions for CHWs. Therefore, training can improve coordination, planning, and communication among partners as mentioned in *Recommendation 2*. Many community participants commented that there were ample training opportunities (S2), but that training should be “streamlined” and “cooperative” (W4). Training should also be incentivized, hands-on, and peer led (O1).

Although participants named training as critical, the literature on the effectiveness of CHW training interventions is still inconclusive. Adams et al. (2021) systematically reviewed training-based interventions to expand the role and increase the capacity of CHWs in African American and Latinx communities. While CHW interventions were effective in improving health outcomes, training-based interventions targeting CHWs were not (Adams et al., 2021). The lack of demonstrable effectiveness of training interventions for CHWs may, however, be explained by weaknesses in study design. Training-based interventions should be considered as emerging strategies and should be further empirically evaluated (Adams et al., 2021).

Alvillar et al. (2011) recommended standardizing training to further define CHW roles and functions. Stupplebeen et al. (2019) found that a lack of a clear career pathway is an obstacle for professional advancement. Standardizing training would be a step toward creating a clear career pathway for CHWs and can also increase CHW recognition and support (Perry et al., 2021; Raffo et al., 2017). Raffo et al. (2017) and Findley et al. (2014) recommended that organizations define interventions suitable for employing CHWs, outline a career pathway for them, and invest in training to further professionalize the CHW workforce. Furthermore, the 2017 CDC report titled *What Evidence Supports State Laws to Establish Community Health Worker Scope of Practice and Certification?* strongly recommended state policies which “establish a certification process for CHWs, by describing education, training, core competencies, reimbursement requirements, and inclusion of CHWs in certification development (Gilchrist et al., 2017).” As of 2016, 16 states had passed CHW scope of practice policies (Gilchrist et al., 2017). Such policies can develop the CHW workforce, standardize evaluation approaches, further define CHW roles and functions, establish more sustainable funding through reimbursement mechanisms, and integrate CHWs more deeply into public health services (Gilchrist et al., 2017).

More firmly codifying the role of CHWs in the public health workforce may mitigate the lack of respect and recognition they face for having lower education and standing within their organizations. On the other hand, professionalizing CHWs may put them at a disadvantage. Participants in the present analysis pointed out that current remuneration for CHWs is not suitable for a qualified professional (T4) and CHW work is susceptible to limited funding (T1), implying that professionalization may dry up available jobs. Professionalization may also raise the threshold to become a CHW (T4), limiting the number of people interested or qualified for the work. In addition, professionalization runs the risk of making CHWs less relevant and connected to the local communities CHWs serve (Schleiff et al., 2021). Therefore, it is necessary to establish the structural conditions outlined in *Recommendations #1 to #4* prior to professionalizing the field to guarantee fair work conditions, wages, and benefits commensurate with CHW education and experience.

## Limitations

There are two limitations of the present SWOT analysis. The first limitation is that a SWOT analysis is a “snapshot” of the *strengths*, *weaknesses*, *opportunities*, and *threats* evident at the time of analysis, and thus, cannot capture how conditions change. Furthermore, the CHW ecosystem is constituted by multiple, dynamic levels of influence, ranging from structural, political, community, organizational, and individual level factors. While our participants highlighted concerns they perceived as relevant, important, and changeable, it is impossible to represent every influence imaginable by applying a rapid community assessment activity such as a SWOT analysis. Given the limited scope of this SWOT analysis, an environmental scan and community health assessment could deepen and nuance these results and overcome this limitation. It is important to repeatedly revisit the results of this SWOT analysis and incorporate the results into future research and planning activities.

The second limitation of the present SWOT analysis is that it does not prioritize issues or solve the problems identified. While we helped prioritize issues by ranking SWOT analysis results with participants, further data collection can provide more information for community partners to strategically plan, set priorities, and work together. The broad recommendations above, therefore, serve as a springboard for future data collection and planning but are themselves limited given the scope of a SWOT analysis. Researchers, practitioners, and policymakers in other locations and contexts should consider which of our recommendations apply to their efforts supporting their local CHW workforce.

## Conclusions

Public health service fragmentation and challenges in the health system, especially during the COVID-19 pandemic, posed major obstacles to the CHW workforce. Our findings correspond to the literature which demonstrates that CHWs, if supported, play an instrumental role in managing respiratory diseases and other chronic conditions (Parekh et al., 2019). To assist CHW stakeholders to strengthen their infrastructure and prepare for future public health crises, we conducted a SWOT analysis with a diverse group of workers in the field. Based on our analysis, we laid out five broad recommendations CHW stakeholders can consider as they collaboratively plan and implement future work. Future research can further explore mechanisms which increase capacity for CHWs, improve industry collaboration, and raise wages and standards for an often-under-resourced group of public health workers. As trusted intermediaries between members of underserved communities and health service agencies, CHWs are a critical public health resource to the most vulnerable and hard-to-reach communities. Researchers, practitioners, and policymakers should create a sustainable CHW ecosystem that avails CHWs of a livable wage and a career path with opportunities for professional advancement.
